# Emergency Department Visits for Cannabis Hyperemesis Syndrome Among Adolescents

**DOI:** 10.1001/jamanetworkopen.2025.20492

**Published:** 2025-07-14

**Authors:** Michael S. Toce, Michael C. Monuteaux, Michael D. Fishman, Joel D. Hudgins

**Affiliations:** 1 Division of Emergency Medicine, Boston Children’s Hospital, Boston, Massachusetts; 2 Harvard Medical Toxicology Program, Boston Children’s Hospital, Boston, Massachusetts; 3 Department of Pediatrics, Harvard Medical School, Boston, Massachusetts

## Abstract

This cross-sectional study assesses trends in emergency department encounters for cannabis hyperemesis syndrome among adolescents.

## Introduction

Cannabis remains the most commonly used illicit substance among adolescents, with evidence showing that recreational cannabis legalization (RCL) with the presence of an active dispensary is associated with an increase in the frequency of use among active users.^1,2^ An increasingly recognized complication of chronic cannabis use is cannabis hyperemesis syndrome (CHS), a variant of cyclic vomiting syndrome that arises in susceptible individuals with chronic cannabis use.^3^ This study aims to assess trends in emergency department (ED) encounters for CHS among adolescents, stratified by state RCL status.

## Methods

This was a retrospective cross-sectional study of adolescent (13 to 21 years) ED encounters for CHS between January 1, 2016, to December 31, 2023. Data were obtained from the Pediatric Health Information System (PHIS) database. ED encounters were identified based off of a principal code for nausea or vomiting, and at least 1 secondary diagnostic code matching a cannabis-related concern (eMethods in Supplement 1).^4^ The presence of RCL with an active dispensary was defined as the exposure (eMethods in Supplement 1). Our primary outcome was the yearly frequency of CHS encounters per 1 000 000 ED encounters in state-years with and without RCL. To assess temporal trends, we estimated a series of population-averaged negative binomial regression models. Effect size estimates were expressed as incident rate ratios (IRR) and 95% CIs (eMethods in Supplement 1). The institutional review board at Boston Children’s Hospital determined that this study was exempt from review because it used only deidentified patient data and met criteria for waiver of informed consent. The study followed the Strengthening the Reporting of Observational Studies in Epidemiology (STROBE) reporting guideline. Data were analyzed from September 2024 to October 2024 using STATA version 18.0 (StataCorp).

## Results

Between January 1, 2016, to December 31, 2023, we identified 4571 encounters of CHS, with a median (IQR) age of 17.0 (16.0-18.0) years (Table). In the entire cohort, CHS encounters increased by 49.0% (95% CI, 43.7%-54.6%) per year, from 160.4 (95% CI, 113.2-207.6) per 1 000 000 ED visits in 2016 to 1968.3 (95% CI, 1580.1-2356.5) per 1 000 000 ED visits in 2023. Encounters for CHS increased by 32.5% (95% CI, 24.6%-40.9%) per 1 000 000 ED encounters per year in states with RCL and by 49.3% (95% CI, 42.6%-56.3%) in states without RCL (Figure). The overall rate per 1 000 000 ED encounters for CHS in states with and without RCL were 1909.5 (95% CI, 1572.9-2246.2) per 1 000 000 ED encounters and 834.0 (95% CI, 698.2-969.0) per 1 000 000 ED encounters, respectively.

**Table.** Demographic and Clinical Characteristics of Adolescents and Young Adults Treated in the Emergency Department (ED) for Cannabis-Hyperemesis Syndrome

Demographic and clinical characteristics	Total sample (n = 4571)	Encounters^a^	
		Non-RCL (n = 3005)	RCL (n = 1566)
Age categories, y			
13-18	3684 (80.6)	2490 (82.9)	1194 (76.2)
19-21	887 (19.4)	515 (17.1)	372 (23.8)
Age, median (IQR), y	17.0 (16.0-18.0)	17.0 (16.0-18.0)	17.0 (16.0-18.0)
Sex			
Female	2888 (63.2)	1946 (64.8)	942 (60.2)
Male	1682 (36.8)	1058 (35.2)	624 (39.8)
Race or ethnicity			
Hispanic	1298 (28.4)	582 (19.4)	716 (45.7)
Non-Hispanic Black	1132 (24.8)	913 (30.4)	219 (14.0)
Non-Hispanic White	1769 (38.7)	1314 (43.7)	455 (29.1)
Other^b^	372 (8.1)	196 (6.5)	176 (11.2)
Insurance status			
Public	2878 (63.0)	1800 (59.9)	1078 (68.8)
Private	1338 (29.3)	917 (30.5)	421 (26.9)
Other	355 (7.8)	288 (9.6)	67 (4.3)
Patient urbanicity (based on zip code)			
Not urban	266 (5.9)	190 (6.4)	76 (4.9)
Urban or suburban	4264 (94.1)	2792 (93.6)	1472 (95.1)
Overall child opportunity level			
Very low	1433 (31.4)	980 (32.7)	453 (28.9)
Low	935 (20.5)	587 (19.6)	348 (22.2)
Moderate	789 (17.3)	558 (18.6)	231 (14.8)
High	774 (17.0)	506 (16.9)	268 (17.1)
Very high	634 (13.9)	369 (12.3)	265 (16.9)
Disposition			
Discharged	2666 (58.3)	1654 (55.0)	1012 (64.6)
Floor or observation unit	1878 (41.1)	1336 (44.5)	542 (34.6)
ICU	27 (0.6)	15 (0.5)	12 (0.8)

Abbreviations: ICU, intensive care unit; RCL, recreational cannabis legalization.

^a^ Non-RCL encounters are ED visits that occurred at hospitals located in states without recreational cannabis legislation in effect; RCL encounters are ED visits that occurred at hospitals located in states with recreational cannabis legislation in effect.

^b^ Other includes Asian, multiple, Other, and unknown.

**Figure.**
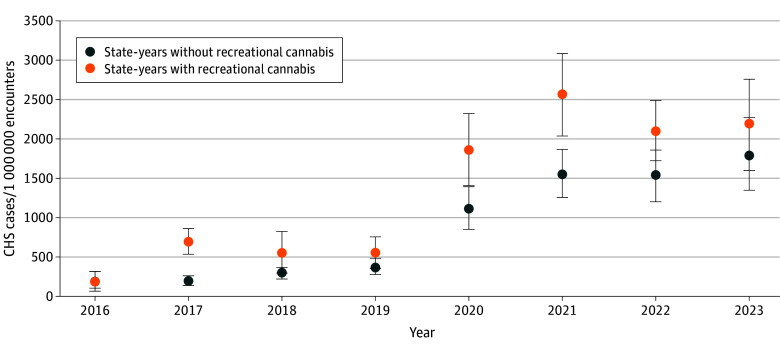
Estimated Annual Rates of ED Encounters for Cannabis Hyperemesis Syndrome (CHS) in State-Years With and Without Recreational Cannabis, January 2016 to December 2023 Seven states changed status (from no recreational cannabis to recreational cannabis) during the study period, comprising 17 of the 48 hospitals (35%) in the sample.

## Discussion

In this cross-sectional analysis of adolescents aged 13 to 21 years, ED visits for CHS increased more than 10-fold during the study. Increases were observed regardless of the state’s RCL status. Limitations of the study include use of an administrative database that may not be generalizable to other populations, and the use of a combination of *International Statistical Classification of Diseases and Related Health Problems, Tenth Revision* codes to identify cases.

CHS is poorly described in the adolescent population.^5,6^ A study that described trends in CHS among youth 15 to 24 years between 2006 to 2020 found that ED visits increased from 6.8 per 1 000 000 population in 2006 to 173.2 per 1 000 000 population in 2020, corresponding to a mean annual increase of 28.1% (95% CI, 26.7%-30.2%).^6^ In our sample, we found an increase of 49.0% per year, which likely reflects a different study period and different patient population. A study examining the association of cannabis legalization with ED encounters for CHS in Ontario, Canada, found that commercialization of cannabis during the COVID-19 pandemic was not associated with increased ED visits for CHS in adolescents 15 to 18 years, however, cases increased throughout the study period.^4^

In conclusion, we found that ED encounters for CHS are increasing both in states with and without RCL, although there were a greater number of cases in RCL states. Additional studies are needed to assess the association of RCL on adolescent CHS to help inform policy makers who are considering RCL.

## Notes

Supplement 1.
eMethods.

Supplement 2.
Data Sharing Statement
