# Supplementary material for: Emergency Department Visits for Cannabis Hyperemesis Syndrome Among Adolescents
Source: JAMA Netw Open. 2025 Jul 14;8(7):e2520492. doi: 10.1001/jamanetworkopen.2025.20492 (PMC12260988; doi:10.1001/jamanetworkopen.2025.20492)
Supplement: Supplement 1. — eMethods. [file jamanetwopen-e2520492-s001.pdf]

Supplemental Online Content

Toce MS, Monuteaux MC, Fishman MD, Hudgins JD. Emergency department visits for cannabis hyperemesis syndrome among adolescents. *JAMA Netw Open*. 2025;8(7):e2520492. doi:10.1001/jamanetworkopen.2025.20492

eMethods.

This supplemental material has been provided by the authors to give readers additional information about their work.

© 2025 Toce MS et al. *JAMA Network Open*.

eMethods.

Identification of ED Encounters for Cannabis Hyperemesis Syndrome in the PHIS Database

The Pediatric Health Information System (PHIS) database is an administrative database that contains inpatient, emergency department, ambulatory surgery and observation encounter-level data from over 50 not-for-profit, tertiary care pediatric hospitals in the United States.[1] These hospitals are affiliated with the Children's Hospital Association (Lenexa, KS). Data quality and reliability are assured through a joint effort between the Children's Hospital Association and participating hospitals. For the purposes of external benchmarking, participating hospitals provide discharge/encounter data including demographics, diagnoses, and procedures. Nearly all of these hospitals also submit resource utilization data (e.g., pharmaceuticals, imaging, and laboratory) into PHIS. Data are de-identified at the time of data submission, and data are subjected to a number of reliability and validity checks before being included in the database. For this study, data from 48 hospitals were included. The Institutional Review Board at Boston Children's Hospital determined that this study was exempt from review as it utilized only deidentified patient data and met criteria for waiver of informed consent. Data analysis took place between October 1 and November 1, 2024. The study adhered to the Strengthening the Reporting of Observational Studies in Epidemiology (STROBE) guideline.

We identified ED encounters for patients aged 13-21 years who presented to the ED of a PHIS hospital with an *International Classification of Diseases, Tenth Revision, Clinical Modification (ICD-10-CM)* principal code for nausea and/or vomiting (i.e., any code beginning with *R11*), and at least one secondary diagnostic code matching cannabis abuse/dependence/poisoning (F12.1 Cannabis abuse, F12.2 Cannabis dependence, F12.9 Cannabis use, unspecified, T40.7 Poisoning by, adverse effect of and underdosing of cannabis [derivatives]). This strategy has been used previously in the literature as cannabis hyperemesis syndrome (CHS) does not have a primary ICD-10-CM code.[2–4] Patients with complex chronic conditions were excluded.[5]

Data Sources for State-level Recreational Cannabis Legislation

The presence of recreational cannabis legislation (RCL) with an active dispensary was defined as the exposure. Information on state-level cannabis legalization status was obtained from the National Conference of State Legislatures.[6] These data indicated whether a state had an open dispensary, and if so, the date when dispensary activity began. From these data, we determined if/when a state had active dispensaries at the state-year level. A year was considered "positive" if there was an open dispensary prior to July 1, and "negative" if antecedent. Using these data, we defined a binary RCL variable as positive if an encounter occurred in a state after the authorization of RCL and there were open and functional recreational cannabis dispensaries in the state, and negative otherwise.

Demographic, Clinical Variables, and Analytical Methods

For all encounters, data were extracted on age, sex, race/ethnicity (extracted from the electronic medical record with reporting method varying by participating institution), insurance status, urbanicity (i.e., a binary classification based on the patient zip code), Child Opportunity Index, and disposition.[7] We characterized the demographic and clinical features of our sample using frequencies and proportions for categorical variables and medians with interquartile ranges for continuous variables.

To assess temporal trends in the rate of CHS diagnoses, we estimated a series of population-averaged negative binomial regression models at the hospital-year level, with the number of CHS cases as the dependent variable and time (in years) as the independent variable, with the log of the total number of ED visits in the sample age range at the hospital-year level as the offset (coefficient constrained to 1). To estimate annual rates, time was modeled as a set of indicator variables, with 2016 set as the referent. To estimate the average annual change in CHS diagnosis rates, time was modeled as a continuous covariate. All models utilized an exchangeable correlation structure and robust standard errors (i.e., sandwich estimators, which produce standard errors that are robust to misspecification of the working correlation matrix).[8] Separate models were estimated for hospital-years with vs without RCL. Effect

© 2025 Toce MS et al. *JAMA Network Open*.

estimates were expressed as incident rate ratios (IRR) and 95% confidence intervals. All analyses were conducted with STATA 18.0 (College Station, TX).

The geographic distribution of the PHIS hospitals (48 hospitals across 26 states) coupled with the state-by-state timing of RCL implementation prevented us from performing an analysis that directly examined the association of RCL implementation with the rate of ED encounters for CHS. That is, most states (and thus most hospitals and most of our ED encounters) were classified as either RCL or non-RCL during the entire study period. Specifically, 17 of the 26 states (containing 29 [60%] of our hospitals) did not have an RCL in effect throughout the entire study period, while another 2 states (containing 2 [4%] of our hospitals) had an RCL in effect throughout the entire study period. Only 7 states (containing 17 [35%] of our hospitals), implemented RCL during the study period. At the encounter level, 3,005 (66%) encounters occurred at hospitals located in states without recreational cannabis legislation in effect, while 1,566 (34%) encounters occurred at hospitals located in states with recreational cannabis legislation in effect. More specifically, 2,558 (56%) cases occurred at hospitals located in states that never implemented RCL during the study period, 1,674 (37%) cases occurred at hospitals located in states that had RCL in effect throughout the entirety of the study period, and only 339 (7%) cases occurred at hospitals located in states that implemented RCL during the study period. A methodologically sound assessment of the hypothesis that RCL implementation increases the risk for CHS ED visits with an observational design would require observations not present in our data. That is, we lack the data to adequately estimate pre-policy temporal trends (precluding an interrupted time series approach). We also lack states providing sufficient pre- and post-implementation observations, along with states without implementation over the same period (precluding a difference-in-differences approach). In sum, these data do not provide sufficient observations measured under the requisite conditions (with respect to exposure and time) to provide a valid assessment of this hypothesis, given current methodological standards for the evaluating state-level policies on clinical outcomes.

© 2025 Toce MS et al. *JAMA Network Open*.

Reference:

1. Children’s Hospital Association. Leverage clinical and resource utilization data. Accessed November 1, 2022. https://www.childrenshospitals.org/phis

2. Myran DT, Roberts R, Pugliese M, Taljaard M, Tanuseputro P, Pacula RL. Changes in Emergency Department Visits for Cannabis Hyperemesis Syndrome Following Recreational Cannabis Legalization and Subsequent Commercialization in Ontario, Canada. *JAMA Netw Open*. 2022;5(9):e2231937. doi:10.1001/jamanetworkopen.2022.31937

3. Wang GS, Buttorff C, Wilks A, Schwam D, Tung G, Pacula RL. Changes in Emergency Department Encounters for Vomiting After Cannabis Legalization in Colorado. *JAMA Netw Open*. 2021;4(9):e2125063. doi:10.1001/jamanetworkopen.2021.25063

4. Costales B, Lu Y, Young-Wolff KC, et al. Prevalence and trends of suspected cannabinoid hyperemesis syndrome over an 11-year period in Northern California: An electronic health record study. *Drug Alcohol Depend*. 2024;263:112418. doi:10.1016/j.drugalcdep.2024.112418

5. Feudtner C, Feinstein JA, Zhong W, Hall M, Dai D. Pediatric complex chronic conditions classification system version 2: Updated for ICD-10 and complex medical technology dependence and transplantation. *BMC Pediatr*. 2014;14(1):1-7. doi:10.1186/1471-2431-14-199

6. State Medical Cannabis Laws. National Conference of State Legislatures. Accessed July 27, 2023. https://www.ncsl.org/health/state-medical-cannabis-laws

7. Child Opportunity Index. Accessed February 1, 2023. https://www.diversitydatakids.org/child-opportunity-index

8. Fitzmaurice GM, Laird NM, Ware JH. *Applied Longitudinal Analysis*. Second edition. Wiley; 2011.

© 2025 Toce MS et al. *JAMA Network Open*.
