# Supplementary material for: Emergency Department Visits for Cannabis Hyperemesis Syndrome Among Adolescents
Source: JAMA Netw Open. 2025 Jul 14;8(7):e2520492. doi: 10.1001/jamanetworkopen.2025.20492 (PMC12260988; doi:10.1001/jamanetworkopen.2025.20492)
Supplement: Supplement 2. — Data Sharing Statement [file jamanetwopen-e2520492-s002.pdf]

# Data Sharing Statement

Toce. Emergency Department Visits for Cannabis Hyperemesis Syndrome Among Adolescents. *JAMA Netw Open*. Published July 14, 2025.
doi:10.1001/jamanetworkopen.2025.20492

**Data**
**Data available:** No
